# Axonemal Lumen Dominates Cytosolic Protein Diffusion inside the Primary Cilium

**DOI:** 10.1038/s41598-017-16103-z

**Published:** 2017-11-17

**Authors:** Wangxi Luo, Andrew Ruba, Daisuke Takao, Ludovit P. Zweifel, Roderick Y. H. Lim, Kristen J. Verhey, Weidong Yang

**Affiliations:** 1 Department of Biology, Temple University, Philadelphia, Pennsylvania 19122 USA; 2 Department of Cell and Developmental Biology, University of Michigan Medical School, Ann Arbor, MI 48109 USA; 3 Biozentrum and the Swiss Nanoscience Institute, University of Basel, Basel, Switzerland

## Abstract

Transport of membrane and cytosolic proteins in primary cilia is thought to depend on intraflagellar transport (IFT) and diffusion. However, the relative contribution and spatial routes of each transport mechanism are largely unknown. Although challenging to decipher, the details of these routes are essential for our understanding of protein transport in primary cilia, a critically affected process in many genetic diseases. By using a high-speed virtual 3D super-resolution microscopy, we have mapped the 3D spatial locations of transport routes for various cytosolic proteins in the 250-nm-wide shaft of live primary cilia with a spatiotemporal resolution of 2 ms and <16 nm. Our data reveal two spatially distinguishable transport routes for cytosolic proteins: an IFT-dependent path along the axoneme, and a passive-diffusion route in the axonemal lumen that escaped previous studies. While all cytosolic proteins tested primarily utilize the IFT path in the anterograde direction, differences are observed in the retrograde direction where IFT20 only utilizes IFT, and approximately half of KIF17 and one third of α–tubulin utilizes diffusion besides IFT.

## Introduction

The primary cilium is a microtubule-based protrusion on the surface of many eukaryotic cells. It functions as an antenna that receives signals associated with cell growth, metabolism, and mechano- and chemo-sensation both in development and during an organism’s adult life cycle^1–4^. Studies on the ultrastructure of primary cilia have suggested that the entire organelle is 1–9 µm in length^5^ and approximately 250 nm in diameter, within which the 9 + 0 axoneme encompasses an axonemal lumen with a diameter of approximately 160 nm^6^. Defects in ciliary structure and function cause a variety of human diseases called ciliopathies^7^.

Primary cilia contain a unique complement of proteins and lipids that enable them to carry out their unique functions in cell motility and signaling^8,9^. However, they are incapable of synthesizing their own protein and thus nearly 200 unique ciliary proteins need to be trafficked between the cytosol and primary cilia^10^. Intraflagellar transport (IFT) trains, formed with IFT protein complexes and driven by the concerted action of kinesin and dynein motor proteins, have been identified as a major transport mechanism for carrying protein cargos directionally along the axoneme within the primary cilium^11,12^. Originally discovered in *Chlamydomonas reinhardtii* flagella and later found to operate in mammalian cilia^13,14^, the IFT particle is a 17-protein complex organized into A and B subcomplexes^14^. Reliant on ATP, Heterotrimeric Kinesin 2(KIF3A-KIF3B-KAP3) and homodimer kinesins 2(KIF17) transport their cargos in the anterograde direction (from base to tip) and dyneins 2 are responsible for the retrograde transport (from tip to base) in the IFT mechanism^14^.

Interestingly, recent emerging evidence suggested that proteins also undergo diffusion in flagella and primary cilia^15–18^. By using various fluorescence microscopy techniques, transport dynamics of both ciliary membrane and cytosolic proteins have been measured in flagella and primary cilia^15–17,19^. These studies revealed that protein diffusion, interspersing with or becoming independent of the IFT-train directional movements, is a critical transport mode for membrane and cytosolic protein trafficking in flagella and primary cilia. Although the previous work has provided great insight into these two transport modes, a couple of critical questions remain unanswered, such as 1) where the diffusion route localizes and 2) what the spatial relationship is between the IFT train and the diffusion pathways.

However, it is still a technical challenge to answer these questions due to the limitations of current techniques. For example, immunogold-labeling and electron microscopy (EM) have been successfully used to determine the localizations of IFT trains in flagella and primary cilia but this technique cannot capture live transport events^13,20^. Conventional fluorescence microscopy, such as wide-field epifluorescence microscopy, scanning confocal microscopy, and total internal reflection fluorescence (TIRF) microscopy can perform live cell imaging but only with low spatiotemporal resolutions or limitation in imaging depth. Recently, several super-resolution microscopy techniques (STORM, PALM, RESOLFT/STED) have been employed to obtain sub-diffraction images in live cells, but require seconds to minutes to provide approximately 50-nm resolution in 1D or 2D imaging *in vivo* ^21–25^. Furthermore, high-speed super-resolution of 3D imaging is even more challenging for these techniques^26^.

To answer these critical questions in primary cilia, we have developed and employed a high-speed virtual three-dimensional (3D) super-resolution microscopy, termed single-point edge-excitation sub-diffraction (SPEED) microscopy, to determine the 3D spatial location of the diffusion route relative to the IFT train pathway for proteins in live primary cilia. Previously SPEED microscopy has enabled us to successfully distinguish the 3D spatial locations of passive diffusion and facilitated translocation routes through the ~50-nm channel of the nuclear pore complex (NPC) with a high spatiotemporal resolution (8–10 nm and 400 μs) in permeabilized and live cells^27–30^. Although both the NPC and the primary cilium have ultra-structures with a rotational symmetry, they are different organelles and possess different transport mechanisms for macromolecule trafficking. Whether SPEED microscopy could be well-suited for mapping transport routes in primary cilia needs to be tested. Given that the structure of the primary cilium can be simplified as a tube-like physical model, we first validated SPEED microscopy by mapping 3D spatial distribution of dye molecules randomly moving in a glass nanocapillary (GNC). After validation, we then determined the 3D transport routes for various ciliary cytosolic proteins, such as IFT20, a component of the IFT subcomplex B^11^, α-tubulin, a protein in the “cargo” class of the IFT train^17^, and KIF17, a member of the kinesin family of motor proteins^31^. Additionally, exogenous GFP was specifically used to study molecular passive diffusion in primary cilia. Overall, we found that IFT20 only moves directionally along the microtubules by adopting the known IFT train pathway, while unexpectedly up to half of KIF17 and one third of α-tubulin diffuse through the axonemal lumen in addition to their directional movements with the IFT train. Moreover, exogenous GFP only passively diffuses through the axonemal lumen and confirms this new axonemal luminal route.

## Results

### 3D mapping of randomly diffusing molecules inside a glass nanocapillary by SPEED microscopy

In principle, a 3D map of protein transport routes is necessary to fully understand how proteins are transported in primary cilia. Given the sub-micrometer ultrastructure of primary cilium, to our knowledge, it is still formidable to map the 3D locations of transport pathways in live primary cilia. With the successful application of SPEED microscopy in mapping 3D transport routes through the NPC^27–30^, here we expanded the approach to determine 3D spatial locations of transport pathways for various ciliary cytosolic proteins moving within the live primary cilia. However, since the primary cilium possesses a different ultrastructure than the NPC, we set out to first validate SPEED microscopy by using a GNC as a model system.

**Figure 1:**
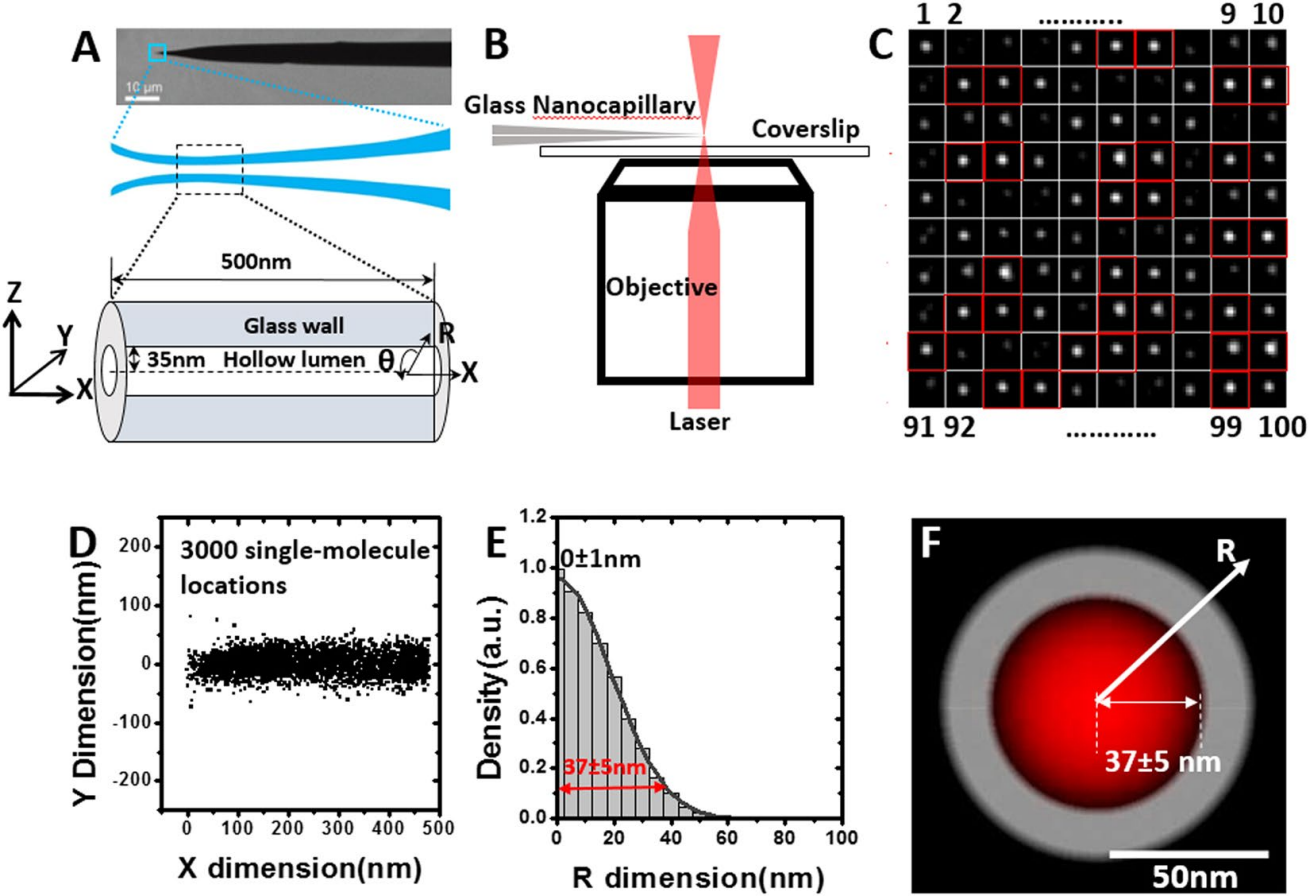
3D mapping of randomly diffusing dye molecules inside a glass nanocapillary (GNC) by SPEED microscopy. (**A**) Determined by HeSTIM, the GNC possesses an inner radius of 35 nm. The spatial locations of individual molecules inside the GNC’s lumen can be represented in a Cartesian (X, Y, Z) or a cylindrical (X, R, θ) coordinate system. (**B**) A simplified schematic of the experimental setup showing a vertical single-point illumination applied on the tip of a GNC. (**C**) Montage of single Alexa Fluor 647 dye molecules randomly diffusing inside the GNC captured by SPEED microscopy at 2 ms per frame. Only those single-molecule spots with a spatial localization precision ≤10 nm (enclosed in red square) were selected to form the 2D super-resolution spatial distribution shown in D. Numbers refer to the frame number. (**D**) 2D super-resolution distribution of dye molecules within the GNC’s lumen. (**E**) By a 2D to 3D transformation algorithm, the spatial probability density distribution of dye molecules inside the GNC’s lumen along the R dimension was obtained. Based on Gaussian function fitting (*e* ^−2^ width), approximately 95% of the molecules inside the GNC’s lumen are contained in a tube-like space with a radius of 37 ± 5 nm. (**F**) Cross-section view of the spatial probability density distribution (red clouds) of dye molecules within the GNC (grey).

First, the dimensions of GNCs used in this study were determined by Helium scanning transmission ion microscopy (HeSTIM), a technique similar to Scanning transmission electron microscopy (STEM) differing mainly in the use of He ions as the illuminating beam^32^. Using this technique, the inner radius of the GNC was determined to be ~35 nm (Fig. 1A). With that parameter in mind, the dimensions of the GNC were re-measured by SPEED microscopy (Fig. 1B). We pumped a solution containing 1-nM Alexa Fluor 647 dye molecules into the inner lumen of the GNC and captured their randomly diffusing movements through the single-point illumination volume (illumination point spread function - iPSF) of SPEED microscopy at a temporal resolution of 2 ms (Fig. 1C). Of note, a vertical iPSF was used rather than an inclined iPSF used previously in the SPEED microscopy setups (Fig. 1B)^27–30^. By single-molecule localization via Gaussian-fitting procedures, thousands of 2D spatial locations of these individual dye molecules were obtained and further filtered to have a spatial localization precision <10 nm (Materials and Methods). Finally, these filtered locations generated a 2D super-resolution distribution for dye molecules diffusing in the GNC (Fig. 1D).

Subsequently, these 2D super-resolution locations were converted to a 3D computed spatial probability density map in the GNC using a transformation algorithm previously developed in our laboratory^27–30^. In general, different 3D spatial distributions of molecules within a tubular lumen will generate different 2D projected images (Fig. S1A and G). It is important to note that either a Cartesian (X, Y, Z) or a cylindrical (X, R, Ɵ) coordinate system can be used to describe a molecule’s position as it travels through the lumen (Fig. S1). Because of a homogenous distribution of randomly diffusing molecules in Ɵ dimension caused by the rotational symmetry of the GNC (Fig. S1E and K), the transformation algorithm between the 2D projected locations (X, Y) in the Cartesian coordinate system and the corresponding 2D cross-section distribution (X, R) in the cylindrical coordinate system is developed (Fig. S1E,F and K,L). As a result, the spatial probability density (***ρ***) in the R dimension for each sub-region along the X dimension of the tube can then be determined (Fig. S1 E and K). Finally, a reconstruction of these spatial probability density maps along the X dimension generates the dimensions of the GNC lumen (Fig. 1E,F).

Using the above 2D to 3D transformation process, SPEED microscopy has enabled us to determine that the GNC has an inner radius of 37 ± 5 nm (Fig. 1E,F), agreeing well with the 35-nm inner radius of the GNC imaged by HeSTIM. Thus, the above tests confirm that SPEED microscopy is well suited to study the 3D diffusion routes for single molecules moving within a tube-like sub-micrometer cavity.

### 3D spatial location of the transport route for IFT20 inside live primary cilia mapped by SPEED microscopy

**Figure 2:**
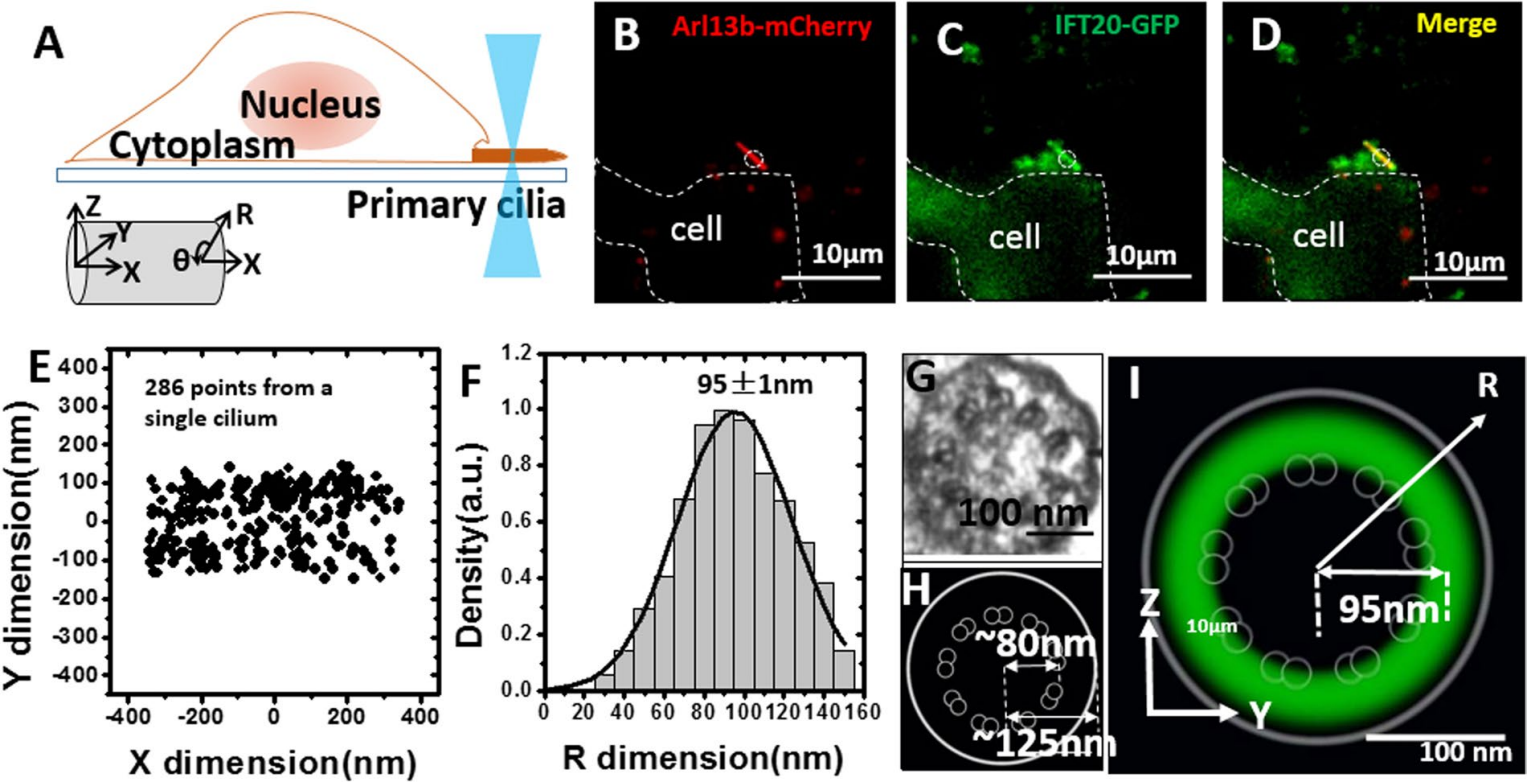
3D spatial location of the transport route for IFT20 inside live primary cilia mapped by SPEED microscopy. (**A**) Schematic figure showing SPEED microscopy illumination of a primary cilium that extends off the side of cell. The Cartesian and the cylindrical coordinate systems are shown. (**B**–**D**) Representative image of (**B**) Arl13b-mCherry and (**C**) IFT20-GFP co-expressed in an NIH3T3 cell and the (**D**) merged. The circle (cyan) indicates the location of single-point illumination of SPEED microscopy on a primary cilium grew on the side of a cell (white dashed lines). Scale bar: 10 μm. (**E**) 2D super-resolution spatial distribution of 286 individual IFT20-GFP locations collected from a single primary cilium. (**F**) By a 2D to 3D transformation algorithm, the spatial probability density distribution of IFT20-GFP inside primary cilia along the R dimension was obtained. Based on Gaussian function fitting, IFT20-GFP primarily locate at a radius of 95 ± 1 nm with a FWHM of 56 ± 5 nm. (**G**) EM data showing the cross-section view of the structure of a primary cilia in 3T3 cells, adapted from reference 6 with permission. (**H**) Schematic indicating the spatial locations of the 9 doublet microtubules and ciliary membrane based on G. (**I**) Cross-section view of the spatial probability density distribution (green clouds) of IFT20-GFP in primary cilia, overlaid with the schematic in H. Scale bar: 100 nm.

With the above validation, we then determined the 3D transport routes for IFT20 in live primary cilia. IFT20, a component of the IFT subcomplex B, has been widely used as a marker for the IFT pathway^11^. In our experiments, we tagged IFT20 with GFP on its C terminus and established a NIH-3T3 cell line stably expressing IFT20-GFP. In addition, to independently localize the primary cilium on the cell surface, we co-expressed Arl13b-mCherry, a widely used ciliary marker protein that associates with the ciliary membrane^33^, in the NIH-3T3 cells (Fig. 2A,B). Moreover, to significantly reduce the background fluorescence or noise from the cytosol, only primary cilia that extended off the side of the cell were selected for imaging (Fig. 2A–D). Finally, we used wide-field epi-fluorescence microscopy to image the entire primary cilium expressing both Arl13b-mCherry and IFT20-GFP, and then switched to SPEED microscopy to track individual IFT20-GFP proteins moving into the iPSF of SPEED microcopy in primary cilia after completely photobleaching of GFP fluorescence down to a background noise level in the iPSF (Fig. 2B–E).

In our experiments, we typically needed two minutes to complete the imaging process and collection of ten single-molecule videos (5,000 frames per video and 2 ms per frame) from a primary cilium, during which the shift of the cilium was controlled to be less than 5 nm for each 10-s video (Methods and Materials). Eventually, hundreds of single-molecule IFT20-GFP locations with a systematical localization precision of <16 nm can be obtained from the primary cilium of a live cell (Fig. 2E, Methods and Materials). Moreover, our simulations based on a cylinder model suggest that a minimum of 250 2D spatial locations is sufficient to generate a reliable 3D spatial map of molecular distribution in the primary cilium (Fig. S2, Materials and Methods). A final determination of IFT20-GFP transport route is based on thousands of single-molecule IFT20-GFP locations collected from ten cilia (Supplementary Fig. S3). Since primary cilia possess a structure with rotational symmetry, the 2D to 3D transformation algorithm was applied to the 2D projected data. Interestingly, the 3D spatial probability density maps of IFT20 indicated a single high density region with a ~56-nm full width at half maximum (FWHM) and peaked at ~95 nm along the radius of primary cilia in eight of ten cilia imaged (Fig. S3). In the other two cilia, a small fraction of IFT20 molecules (<10% of total detected molecules) appeared within the axonemal lumen and shifted off the primary route peaked at ~95 nm (Fig. S3). Therefore, the above 3D spatial distributions of IFT20 suggests that IFT20 primarily moves along a pathway locating at ~95 nm away from the middle axis of primary cilia (Fig. 2F). According to the EM-based ultrastructure of the primary cilium (Fig. 2G,H), this high-density region likely co-localizes with the axonemal microtubules, in agreement with the known location of the IFT route (Fig. 2I). The minor off-peak locations of IFT20 could be assigned to the one or two doublet microtubules in the axoneme that deviate from the radially-symmetric 9 + 0 organization and localize more centrally located in the ciliary shaft^34^.

To test whether IFT20 is moving directionally or randomly along the IFT route, we also determined the transport kinetics for IFT20-GFP based on single-molecule trajectories. In our experiments, a wide-field epi-fluorescence illumination with a ten-fold lower excitation power (1 kW/cm^2^) and a fifty-fold slower frame rate (10 frames per second) than SPEED microscopy imaging was used. Montaged frames show a clear directional movement for IFT20-GFP (Fig. S4A). To quantify the type of movement that IFT20 undergoes, the trajectory was plotted as 2D Mean Square Displacement (MSD) vs. time and fitted with the function, MSD = 4Dt^α^ ^35^. D is the diffusion coefficient, t is time, and the α component represents directional movement (or super-diffusion), passive diffusion, or sub-diffusion if its value is bigger than, equal to, or smaller than 1, respectively^35^. The fitting for the IFT20-GFP trajectory yielded an α value of 1.75, suggesting that IFT20 is conducting directional movement in live primary cilia (Fig. S4). Moreover, analysis of 68 longer single-molecule trajectories of IFT20 (frames >6) yielded an average velocity of 0.30 ± 0.05 µm/s for IFT20-GFP (Fig. S4), which is consistent with the IFT velocity of 0.2–0.7 µm/s previously measured in mammalian cells^11,36^. The above measurements indicated that our approach of combining wide-field epi-fluorescence and SPEED microscopy can reveal both the 3D spatial location of the transport route and the transport kinetics for IFT20 in live primary cilia.

### SPEED microscopy revealed that KIF17 and α-tubulin transport through the axonemal lumen in addition to the IFT pathway in live primary cilia

**Figure 3:**
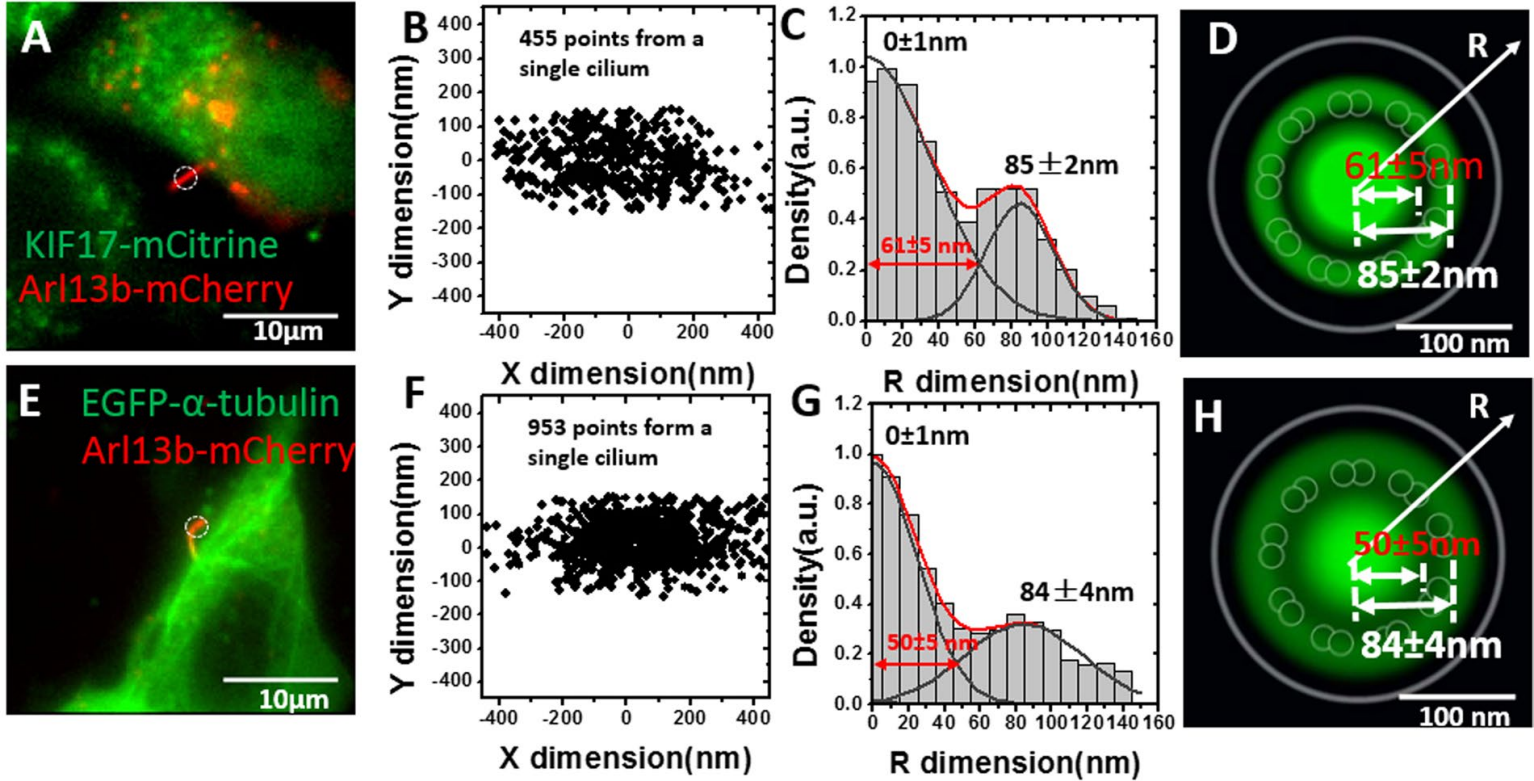
SPEED microscopy revealed that KIF17 and α-tubulin transport through the axonemal lumen in addition to the IFT pathway in live primary cilia. (**A**) Representative image of Arl13b-mCherry and KIF17-mCitrine -expressed in NIH3T3 cells. The circle denotes the laser illumination area of SPEED microscopy. Scale bar: 10 μm. (**B**) 2D super-resolution spatial distribution of 455 individual KIF17-mCitrine locations collected from a single primary cilium. (**C**) By a 2D to 3D transformation algorithm, the spatial probability density distribution of KIF17-mCitrine inside primary cilia along the R dimension was obtained. Based on Gaussian function fitting, two clusters of KIF17-mCitrine locate at radii of ~85 nm and ~0 nm in the cilia. The radius of the central lumen route is 61 ± 5 nm, ranging from the very center to the crossing point of two Gaussian fittings. (**D**) Cross-section view of the spatial probability density distribution (green clouds) of KIF17-mCitrine in primary cilia, overlaid with the schematic in Fig. 2H. Scale bar: 100 nm. (**E**) Representative image of Arl13b-mCherry and EGFP-α tubulin co-expressed in NIH3T3 cells. The circle denotes the laser illumination area of SPEED microscopy. Scale bar: 10 μm. (**F**) 2D super-resolution spatial distribution of 953 individual EGFP-α tubulin locations collected from a single primary cilium. (**G**) By a 2D to 3D transformation algorithm, the spatial probability density distribution of EGFP-α tubulin inside the primary cilia along the R dimension was obtained. Based on Gaussian function fitting, two clusters of EGFP-α tubulin locate at radii of ~84 nm and ~0 nm in the cilia. The radius of the central lumen route is 50 ± 5 nm, ranging from the very center to the crossing point of two Gaussian fittings. (**H**) Cross-section view of the spatial probability density distribution (green clouds) of EGFP-α tubulin in primary cilia, overlaid with the schematic in Fig. 2H. Scale bar: 100 nm.

To obtain a fuller 3D picture of the IFT-directed protein trafficking in primary cilia, two other representative proteins were selected for SPEED microscopy imaging and analysis. KIF17, a member of the kinesin-2 family of motor proteins^37^, and α-tubulin, a structural protein of the axonemal microtubules, were tagged with mCitrine and EGFP respectively. Each was individually co-expressed with Arl13b-mCherry in NIH-3T3 cells for epi-fluorescence imaging and single molecule tracking (Fig. 3A,E). Epi-fluorescence images revealed that KIF17 and α-tubulin have similar concentration in primary cilia and cell body in bulk condition (Fig. 3A,E), which is in contrast to that of IFT20 accumulated in primary cilia (Fig. 2C). These observations agree with previous measurements^11,37^. Following the same imaging and analysis procedures as that of IFT20, we obtained the 3D locations of transport routes for both KIF17 and α-tubulin, based on thousands of single-molecule spatial locations collected from the cilia of ten live cells each (Fig. 3B and F). Different from a single primary transport pathway of IFT20, the 3D spatial locations of transport routes for these two proteins clearly revealed that they have two distinct routes: one route has a peak position at ~85 nm with a width of ~40–70 nm and the other corresponds to a central axonemal luminal region with a peak position at ~0 nm along the radius of primary cilia and a width of ~100–120 nm (Fig. 3C,D and Fig. S3). The former route is similar to that of IFT20 and the location of the IFT pathway along the axonemal microtubules. The differences in the peak positions and widths between these candidates and IFT20 might suggest their different locations in the IFT train. It is noteworthy that in our measurements we noticed either KIF17 or tubulin also possesses an off-zero peak in a one or two cilia that is different from the two representative peaks observed in the majority of cilia (Fig.S3B), which might be possibly caused as well by the displacement of microtubules similar to the aforementioned comments for IFT20. Remarkably, the central pathway reveals a previously uncharacterized pathway for KIF17 and α-tubulin, suggesting that these proteins also transport through the axonemal lumen in addition to the IFT pathway in live primary cilia (Fig. 3C,D and G,H).

Previous studies suggested that IFT20 can be returned to the ciliary base via the dynein-driven IFT transport, while some kinesins and tubulins could randomly diffuse within cilia after they fall off the IFT train in the ciliary shaft and/or at the ciliary tip^17,19,38^. A hypothesis is that the axonemal lumen may function as the route for molecular diffusion within the ciliary shaft. To test this hypothesis and further characterize the usage of the IFT train and the axonemal lumen during protein transport, we completely photobleached fluorescently labeled KIF17 or α-tubulin molecules coming from the ciliary tip before localizing single-molecules in the middle of the ciliary shaft, as shown in Fig. S5A and E. Since no fluorescent protein molecules coming from the tip can be detected, only directional movements from ciliary base to tip and diffusion will be measured (Fig. S6A). In our experiments, the primary cilia were serum-starved for 48 hours before SPEED microscopy imaging, providing sufficient time for primary cilia to stop growing and likely equilibrate the flux of proteins moving between ciliary base and tip (Fig. S6). As shown in Fig. S5–S7, the probability of using the IFT train pathway or the axonemal lumen route was obtained by extracting the spatial probability density distribution in each direction from the summed transport route. For α-tubulin, approximately one third of the molecules employ the axonemal lumen route and the other two thirds follow the IFT train pathway, regardless of the direction of transport (Table 1). Interestingly, approximately only 7% of KIF17 molecules (the percentage represents a density probability of detecting the targeted molecules by including the contributions of both the number and the diffusion coefficient of these molecules) employ the luminal route and 93% rely on the IFT pathway for transport from the ciliary base to tip, while, more than half of KIF17 (~55%) employ the axonemal lumen and the rest use the IFT train route for transport in the tip-to-base direction (Table 1). The primary usage of the IFT train pathway by KIF17 from the ciliary base to tip likely reflects the microtubule-based motility of kinesin motors. KIF17 is the homolog of OSM-3 in C. elegans sensory cilia, OSM-3 can only function as motor in anterograde IFT trains, but it could also be carried as cargo in retrograde transport driven by dynein^38^. The fact that more than half of KIF17 (~55%) returning to the cell body by diffusing through the axonemal lumen suggests that a significant portion of kinesin motors do not undergo dynein-driven retrograde transport, in agreement with previous observations^39^.

**Table 1:** Probability of adopting each transport route for KIF17 and α-Tubulin.

Transport route	KIF17			α-Tubulin		
	Summed*	Base to tip	Tip to base	Summed*	Base to tip	Tip to base
Axonemal lumen	31 ± 1%	7 ± 1%	55 ± 1%	30 ± 1%	32 ± 1%	29 ± 1%
IFT	69 ± 1%	93 ± 1%	45 ± 1%	70 ± 1%	68 ± 1%	71 ± 1%

*Summed indicates condition of simultaneous bi-directional transport within the cilium.

### GFP passively diffuse through the axonemal lumen

**Figure 4:**
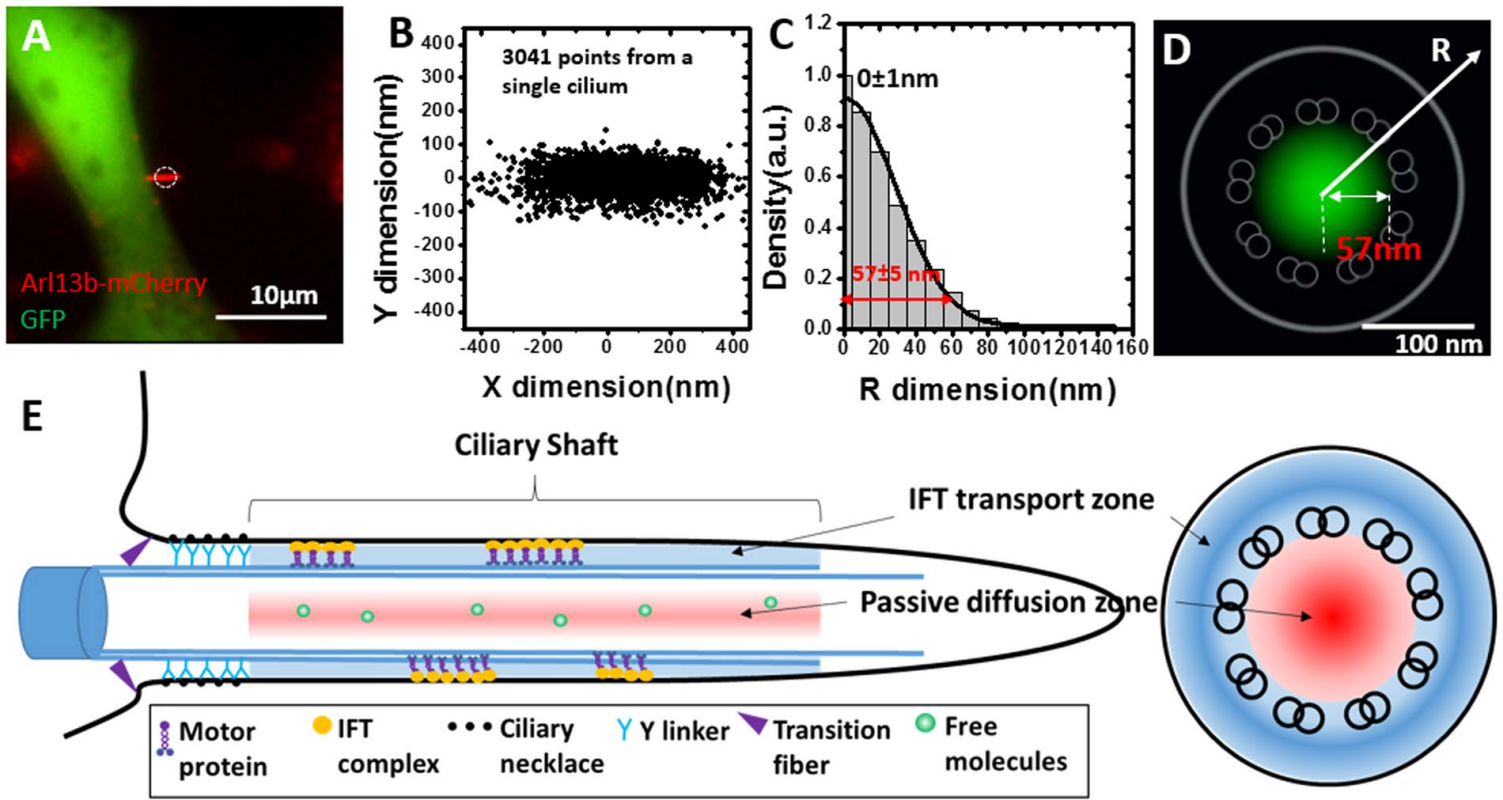
GFP passively diffuse through the axonemal lumen. (**A**) Representative image of Arl13b-mCherry and GFP co-expressed in NIH3T3 cells. The circle denotes the laser illumination area of SPEED microscopy. Scale bar: 10 μm. (**B**) 2D super-resolution spatial distribution of 3041 individual GFP locations collected from a single primary cilium. (**C**) By a 2D to 3D transformation algorithm, the spatial probability density distribution of GFP inside the primary cilia along the R dimension was obtained. Based on Gaussian function fitting (e^−2^ width), approximately 95% of GFP molecules inside the axonemal lumen are contained in a tube-like space with a radius of 57 ± 5 nm. (**D**) Cross-section view of the spatial probability density distribution (green clouds) of GFP in primary cilia, overlaid with the schematic in Fig. 2H. Scale bar: 100 nm. (**E**) Transport model schematic showing the spatial locations of the IFT transport pathway and the passive diffusion route for cytosolic proteins in shaft of primary cilia with a side-view (left) and a cross-section view (right).

Powered by ATP, motor proteins transport their cargos in either the anterograde or retrograde direction by directly interacting with axonemal microtubules in the IFT mechanism. Although the axonemal lumen occasionally can hold one or two doublet microtubules due to the disorganized arrangement of 9 + 0 doublet microtubule structure in the ciliary shaft of NIH 3T3 cells, previous EM has not shown that IFT trains could exist in the luminal region^6,34^. Thus, a remaining question is how KIF17 and α-tubulin transport through the axonemal lumen if not adopting the IFT mechanism. To answer this question, we utilized GFP as a candidate diffusing protein and determined its moving pattern and diffusion route within the shaft of primary cilia (Fig. 4A). Interestingly, we found that 100% of GFP molecules moved through the axonemal luminal region in the shaft of primary cilia (Fig. 4B–D), suggesting that the axonemal lumen functions as a passive diffusion route for these proteins. To further test this suggestion, we also determined the diffusion coefficient of GFP by analyzing hundreds of GFP’s single-molecule trajectories in the ciliary shaft of live primary cilia. As shown in Fig. S3E, a linear fitting by the MSD function generated a diffusion coefficient of 3.6 µm^2^/s for GFP, agreeing well with previous determinations^17^. Therefore, we concluded that there are two distinct transport routes for cytosolic proteins moving inside primary cilia: one is the microtubule-directed IFT pathway, and the other is the IFT-independent passive-diffusion route located in the axonemal lumen (Fig. 4E).

## Discussion

The 3D spatial mapping of the cytosolic protein transport routes in live primary cilia yielded new insights into the protein transport mechanisms within primary cilia. First, SPEED microscopy can distinguish the 3D spatial locations of distinct transport routes in sub-micrometer organelles with rotational symmetry, such as the NPC and the primary cilium. Although numerous 3D single-particle tracking techniques have been developed in the past years^40^, it is still formidable to map 3D locations of pathways in both the NPC and the primary cilium in live cells. We demonstrated here that a combination of 2D super-resolution imaging and a 2D to 3D transformation algorithm can provide an alternative high-speed 3D mapping approach to determine the transport pathways in these organelles *in vivo*. We expect the approach to be expanded to investigate more sub-cellular organelles with rotational symmetry, such as mitochondria, peroxisome, and endosome. Second, SPEED microscopy indicates that the majority of proteins undergoing free diffusion within the shaft of the cilium largely localize to the axonemal lumen. Although the IFT train and the diffusion modes have been suggested to be responsible for protein trafficking inside the primary cilia before, the relative spatial relationship between these two transport routes remained unresolvable in previous measurements. Previous EM studies have localized IFT trains along the axonemal microtubules but never imaged them inside the lumen of the axoneme^6,41^. Here for the first time we revealed that this space houses the passive diffusion transport route for ciliary cytosolic proteins.

Third, it seems that cytosolic proteins with different destinations and functions in the cilia could adopt different transport routes. For instance, IFT20 is an essential part of the IFT particle and it unlikely disassociates from the IFT particle during the entire IFT train transport within the primary cilia. Thus, as observed, IFT20 remains associated with the IFT train moving directionally along the “highway” of axonemal microtubules. In contrast, GFP is an exogenous protein without any specific interaction or destination inside the cilia. As a result, it is not a component of the IFT trains traveling along the axonemal microtubules and but rather, it randomly diffuses through the axonemal lumen. Interestingly, our data revealed that both the axonemal lumen passive diffusion route and the IFT directional movements are adopted by the kinesin-2 motor KIF17 and the IFT cargo protein α-tubulin. However, the relative contributions of these routes to the transport of KIF17 and α-tubulin differ. For KIF17, the majority take the IFT pathway in the anterograde transport as active carriers, while approximately half of them diffuse through the axonemal lumen in the retrograde direction. For α-tubulin, the majority utilize the IFT transport route in both the anterograde and retrograde directions. Additionally, it is unlikely that diffusing molecules are completely constrained within the axonemal lumen by the loosely arranged doublet microtubules. Instead, our data suggest that some protein molecules diffuse through the central lumen (such as GFP, IFT-independent diffusing tubulin and KIF17) can slightly diffuse into the space between doublet microtubules and ciliary membrane. Collectively, our findings suggest a model for ciliary cytosolic proteins trafficking in primary cilia that the axonemal lumen of primary cilia is a region of passive diffusion, responsible for motor protein recycling and cargo protein transport if needed, while the region between the axonemal microtubules and the ciliary membrane is responsible for directional movement of the IFT complex and associated motor and cargo proteins (Fig. 4E).

Finally, going forward with this model in mind, we are also very interested in speculating on the consequences on other aspects of ciliary protein transport. Although the diffusion pathway and IFT pathway was mapped in the axonemal region, how does protein transport into or out of primary cilia is still unknown. The base of primary cilia has been shown to have several protein modules that are responsible, at least in part, for the proper localization of membrane and membrane-associated proteins. Given that primary cilia are largely signaling organelles which require the proper localization and recycling of membrane and membrane-associated proteins, exploration of these details will have direct benefits to our understanding of diseases such as Bardet-Biedl syndrome and polycystic kidney disease^42^. Moreover, two distinct transport models have been proposed in the transition zone: (1) cilium pore complexes (CPC) provide controls for soluble proteins entry into the primary cilium and (2) the space between Y-shaped linkers and ciliary membrane facilitate entrance of membrane proteins into the primary cilium^43^. By SPEED microscopy, we plan to expand the approach to specifically study the transport routes and kinetics of both cytosolic and membrane proteins in the transition zone of primary cilia as well. Moreover, whether the two distinct transport routes for the tested cytosolic proteins observed in the shaft of primary cilia could be applicable in other model systems is also our research interest. Some previous studies have already provided some hints. For example, in flagellar the microtubule plus-end tracking protein EB1 was shown to transport by diffusion, while axonemal structure protein DRC4 was shown to be carried by anterograde IFT trains as cargo and later diffused back after it reaches the tip^18,44^. Also, different from mammalian primary cilia, heterotrimeric kinesin 2 was reported to be transported solely depending on IFT train instead of diffusion in *C. elegans* sensory cilia^38,45^, while diffusion is the only way for heterotrimeric kinesin 2 to move from tip to base in Chlamydomonas flagellar^46^.

## Materials and Methods

### Manufacture of glass nanocapillary and sample preparation

Glass nanocapillaries (GNCs) were fabricated by the laser-assisted capillary-pulling of quartz micropipettes with an outer diameter of 0.5 mm and an inner diameter of 0.2 mm (Hilgenberg GmbH). The puller (P-2000, Sutter Instruments) exposes the micropipettes to a tunable CO2 (up to 10 W) IR laser (approx. λ = 10 μm) beam with a minimal spot size of 0.1 mm. Quartz glass is heated up to the melting point (approx. 1700 °C) upon irradiation with the laser. Pulling and heating at the same time then leads to a shrinking of the exposed region of the micropipette. A final pull separates the thinned region in its middle resulting in two GNCs with tunable pore diameters ranging from 20 nm to 300 nm. Dimension measurement imaging was performed with a He ion microscope (OrionPlus, Carl Zeiss Microscopy GmbH).

1-nM Alexa Fluor 647 in PBS (pH 7.2, 150 mM NaCl, Gibco) were pumped into GNCs and then mounted on a microinjection stage with the tips immersed in custom-made, PBS containing reservoirs made from Polydimethylsiloxane (PDMS). The GNCs were brought into contact with the edge of the reservoir close to the tip to reduce vibrations during SPEED measurements. This contact lead also to a bending of the GNC tip region such that a small angle of ~5° between the tip and glass slide has been achieved. The distance between the tip and the glass slide was thereby small enough to enable the usage of a 100x oil- immersion objective (UPLSAPO 100x, Olympus).

### Cell lines, cell transfection and plasmids

NIH-3T3 cells were grown in DMEM, high glucose, GlutaMAX Supplement (Life Technologies), 10% fetal bovine serum (Fisher Scientific), and 1% penicillin-streptomycin (Thermo Fisher) and split every 2 days to 40–50% confluency. 48 hours prior to imaging, the cells were transferred to glass bottom dishes and grown in OPTIMEM (Life Technologies) to induce growth of primary cilia. Plasmids of GFP-alpha Tubulin and IFT20-GFP was a gift from Patricia Wadsworth (Addgene plasmid # 12298) and from Gregory J. Pazour (University of Massachusetts Medical School), respectively. Plasmids of Arl13b-mCherry and KIF17-mCitrine were constructed as described previously^37^. Transfection was performed concurrently with induction of primary cilia growth using Transit-LT1 (Mirus) according to the manufacturer’s protocol. For stable cell lines, 150 μg/ml hygromycin B was used for further selection after transfection. Prior to imaging, media was replaced with transport buffer (20 mM HEPES, 110 mM KOAc, 5 mM NaOAc, 2 mM MgOAc, and 1 mM EGTA, pH 7.3).

### Instrumentation

The SPEED microscope included an Olympus IX81 equipped with a 1.4-NA 100×oil-immersion apochromatic objective (UPLSAPO 100×, Olympus), a 35 mW 633 nm He-Ne laser (Melles Griot), 50 mW solid state 488-nm and 561-nm lasers (Coherent), an on-chip multiplication gain charge-coupled-device camera (Cascade 128 + , Roper Scientific) and the Slidebook software package (Intelligent Imaging Innovations) for data acquisition and processing. For individual channel imaging, GFP (or mCitrine), mCherry, and Alexa Fluor 647 were excited by 488 nm, 561 nm, and 633 nm lasers, respectively. The fluorescence emissions were collected by the same objective, filtered by a dichroic filter (Di01- R405/488/561/635-25 × 36, Semrock) and an emission filter (NF01- 405/488/561/635-25 × 5.0, Semrock) and imaged with the above CCD camera operating at 500 Hz.

### Localization precisions of isolated fluorescent spots

The localization precision for moving fluorescence molecules was defined as how precisely the central point of each detected fluorescent diffraction-limited spot was determined. The influence of particle motion during image acquisition should be considered in the determination of localization precision. In detail, the localization precision for moving substrates (σ) was determined by an algorithm of

$$\sigma =\sqrt{F[\frac{16({s}^{2}+{a}^{2}/12)}{9N}+\frac{8\pi {b}^{2}{({s}^{2}+{a}^{2}/12)}^{2}}{{a}^{2}{N}^{2}}]}$$

where F is equal to 2, N is the number of collected photons, a is the effective pixel size of the detector, b is the standard deviation of the background in photons per pixel, and $s=\sqrt{{s}_{0}^{2}+\frac{1}{3}D{\rm{\Delta }}t}$, s_0_ is the standard deviation of the point spread function in the focal plane, D is the diffusion coefficient of substrate in the primary cilia, and Δt is the image acquisition time^47–50^.

In our experiments, for moving molecules inside the GNC and primary cilia (captured within ~300 nm in the focal plane of SPEED microscopy), the localization precision is calculated to be <10 nm and <14 nm respectively, based on the above equations and the parameters determined experimentally (N > 1500 for protein candidates in primary cilia and N > 3000 for dyes in the GNC, a = 240 nm, b ≈ 2, s0 = 150 ± 50 nm, D is in the range of 1–4µm^2^/s for the tested protein substrates and 4 µm^2^/s for dyes in the GNC). Therefore, the overall tracking precision for labeled proteins moving in live primary cilia is <16 nm.

### Calculation of diffusion coefficient

Calculation of the diffusion coefficient was performed by first plotting each trajectory (>6 frames) on a Mean Square Displacement (MSD) vs. Time (t) plot. The data was fitted with the function MSD = 4Dt^α^, D is the diffusion coefficient; α represents directional movement (or super-diffusion), passive diffusion or sub-diffusion if its value is bigger than, equal to, or smaller than 1 respectively. Velocity was calculated only for directionally moving molecules.

### Transformation algorithms and image processing

The detailed deconvolution process used to compute the 3D spatial probability density maps of particles transiting through the NPC was described in our previous publications^27^ and demonstrated again here in Fig. S1. In short, the 3D spatial locations of molecules transiting through the shaft of primary cilia can be considered in either Cartesian (X, Y, Z) or cylindrical (X, R, Ѳ) coordinates. In microscopic imaging, the observed 2D spatial distribution of particle localizations is a projection of its actual 3D spatial locations onto the XY plane. The underlying 3D spatial distributions can be recovered by projection of the measured Cartesian (X, Y) coordinates back onto the simplified cylindrical (X, R, constant) coordinates, based on the expected cylindrically symmetrical distribution along the Ѳ direction of the cilia. More detailed information can be found in Fig. S1 and its figure caption. The resulting 3D, surface-rendered visualizations shown in figures and movies were generated with Amira 5.2 (Visage Imaging).

### Gaussian fitting of transport routes

The obtained spatial probability density distribution of moving molecules in GNCs or cilia were first mirrored along R dimension with virtual negative radius values and then fitted by one or multiple Gaussian functions. From the Gaussian fittings, the peak position and the width of transport route were determined. Full width at half maximum (FWHM) or e^−2^ width of a Gaussian function or distance between crossing points of two Gaussian fits was used to describe dimension of transport pathway.

### Determination of cilia movement, orientation and axial position

In our experiments, we typically needed two minutes to complete imaging of the entire cilium and collection of ten single-molecule videos (5,000 frames per video and 2 ms per frame) from a primary cilium. Combination of 48-hour serum-starving cell growth and incubation of cells in transport buffer prior to microscopy imaging made the shift of primary cilia in live cells less than 5 nm during the 10-s detection time for each video. As for the cilia orientation, first epi-fluorescence image of Arl13b-mCherry labeled cilium provided a complete image of entire cilium, which clearly indicated the ciliary base, the ciliary tip and the cilia orientation. Then, the point-illumination of SPEED microscopy generated 2D super-resolution spatial distribution of protein molecules moving within a range of approximately 800 nm along the ciliary axis in the shaft of primary cilia. Consequently, the precise location of the middle axis of a primary cilium was obtained by determining the peak position of these 2D super-resolution spatial locations in the ciliary radial dimension with fitting of Gaussian function.

### Statistics

Experimental measurements were reported as mean ± standard error of the mean unless otherwise noted.

### Data availability

The data that support the findings of this study are available on request from the corresponding author (W.Y.).

## Electronic supplementary material

Supplementary Information
