# Supplementary material for: Axonemal Lumen Dominates Cytosolic Protein Diffusion inside the Primary Cilium
Source: Sci Rep. 2017 Nov 17;7:15793. doi: 10.1038/s41598-017-16103-z (PMC5693955; doi:10.1038/s41598-017-16103-z)
Supplement: Supplementary file 1 — Supplementary Information [file 41598_2017_16103_MOESM1_ESM.pdf]

Supporting Information for

Axonemal Lumen Dominates Cytosolic Protein Diffusion inside the Primary Cilium

Authors:

Wangxi Luo[*], Andrew Ruba[*], Daisuke Takao[†], Ludovit P. Zweifel[‡], Roderick Y.H. Lim[‡], Kristen J. Verhey[†] and Weidong Yang[*§]

Affiliations:

* Department of Biology, Temple University, Philadelphia, Pennsylvania, 19122 USA

† Department of Cell and Developmental Biology, University of Michigan Medical School, Ann Arbor, MI. 48109 USA

‡ Biozentrum and the Swiss Nanoscience Institute, University of Basel, Basel, Switzerland

§ **Corresponding author:** Weidong Yang, E-mail: weidong.yang@temple.edu

2D to 3D Transformation Algorithms for Molecules if moving through the Central lumen of a Tube

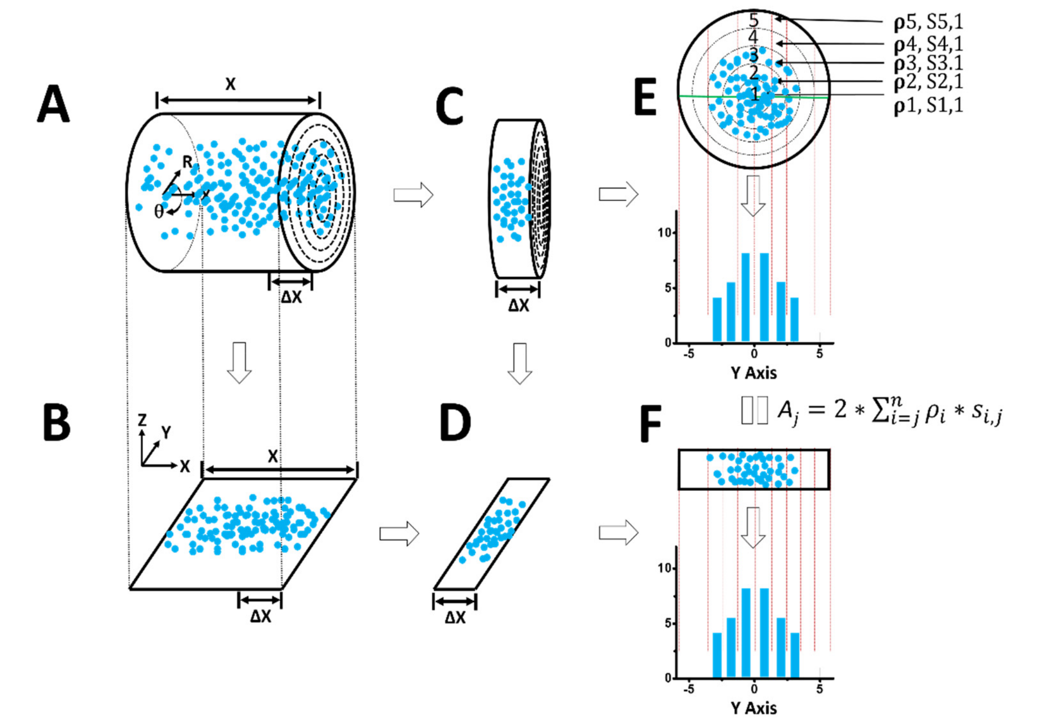

2D to 3D Transformation Algorithms for Molecules if Moving within a Peripheral Region of a Tube

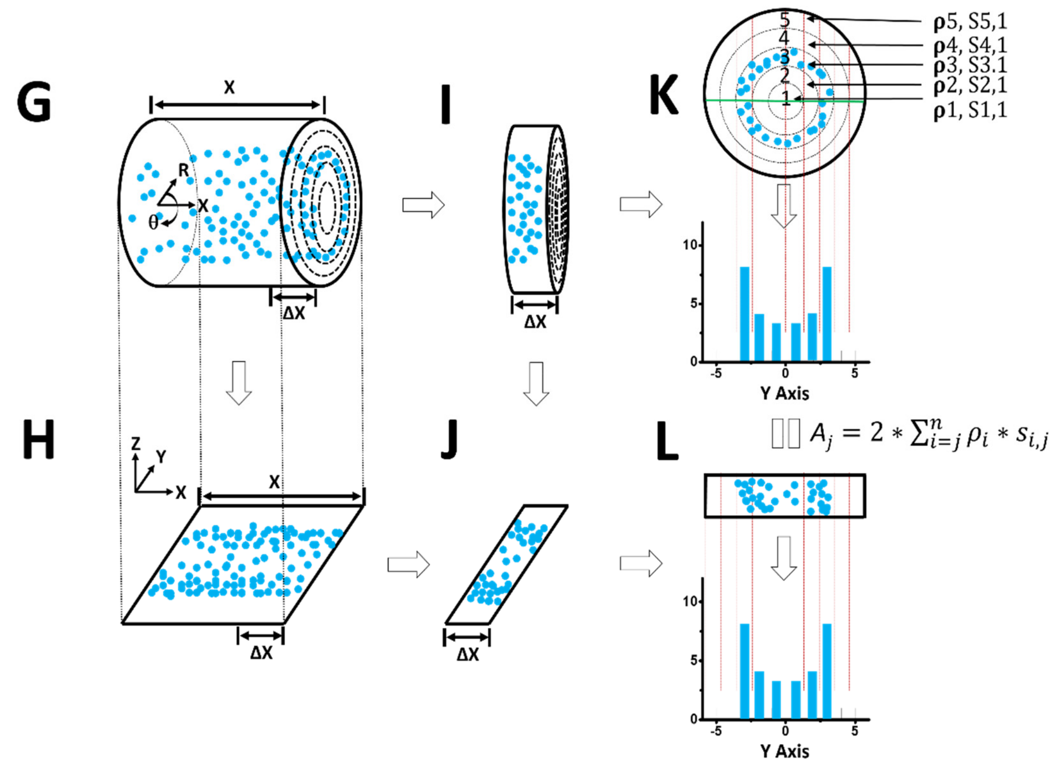

Figure S1 2D to 3D transformation process and algorithms. Schematics demonstrate the 2D to 3D transformation algorithms for molecules, for example, if they diffuse within the central lumen (A-F) or a peripheral region (G-L) of a tube. (A) 3D spatial locations of randomly diffusing molecules inside a tube can be coordinated in a cylindrical coordination system (R, X, ϴ). (B) The 3D molecular locations in A are projected onto a 2D plane in a Cartesian coordination system (X, Y, Z) by microscopy imaging. (C) A very thin slice (Δx) cut from the cylinder in A along x dimension. (D) The 3D spatial locations in the slice shown in C can be projected within a narrow 2D region. (E) Cross-section view of all the locations in the thin slice shown in C. These locations can be grouped into the sub-regions between concentric rings. Given the high-number randomly distributed molecules in the cylinder and the cut very thin slice, the spatial density of locations ($\boldsymbol{\rho}_i$) in each sub-region (S_i) between two neighboring rings will be rotationally symmetrical and uniform. These locations can be further projected into 1D along the Y dimension. If the locations along Y dimension are clustered in a histogram with j columns. The total number of locations in each column (A_j) is equal to $2 * \sum_{i=j}^{n} \rho_i * s_{i,j}$, which can be experimentally measured as shown in (F). (G-L) Similar as the above, the transformation process is presented for molecules diffusing within a peripheral region of a tube. A complete outline of the calculations have been provided in our previous publications (1-4).

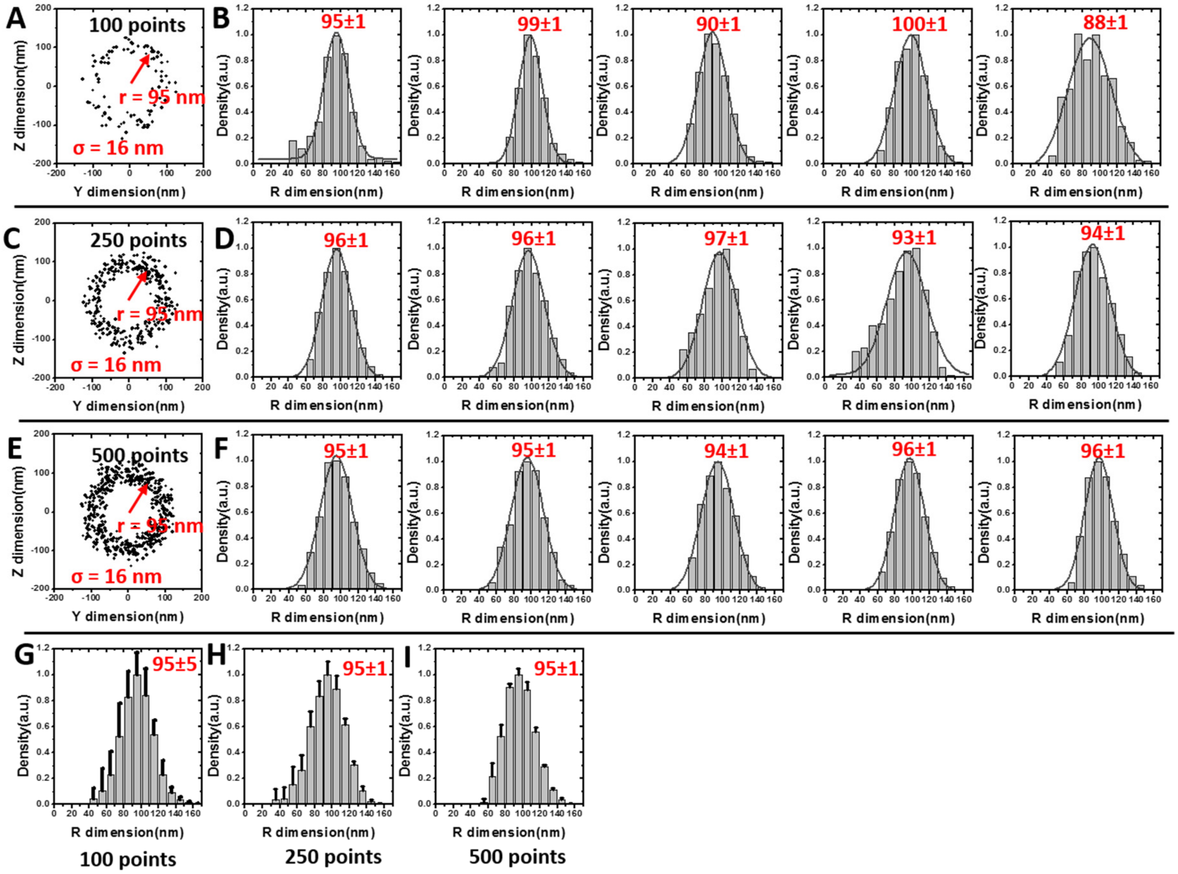

Figure S2 Simulation was used to estimate the minimum number of 2D single-molecule locations to generate a reliable 3D spatial probability density map for the 2D to 3D transformation algorithms. (A) 100 computationally-generated single-molecule locations randomly sampled from a radial density normal distribution centered at 95 nm (corresponding to the primary transport route of IFT20 determined in our experiments). Localization precision of 16 nm was simulated for each single-molecule location by sampling from a normal distribution with σ = 16 nm. As shown in Fig. S1, a very thin slice (Δx) in the X dimension is used for transformation algorithms and therefore the X dimension is not shown in the computationally-generated 2D single-molecule locations. (B) Transformation between Y-dimensional projected

data and 3D R-dimensional density generated histograms of 3D R-dimensional densities for five different simulated data sets (each with 100 points). The number above is the peak position ± fitting error. (C-D) Simulation results based on 250 single-molecule locations. (E-F) Simulation results based on 500 single-molecule locations. (G-I) Average 3D density histograms from 100-, 250- and 500-points simulations respectively. Error bars represent variability in the histogram bin heights while the number above the peak is the average peak position ± standard deviation.

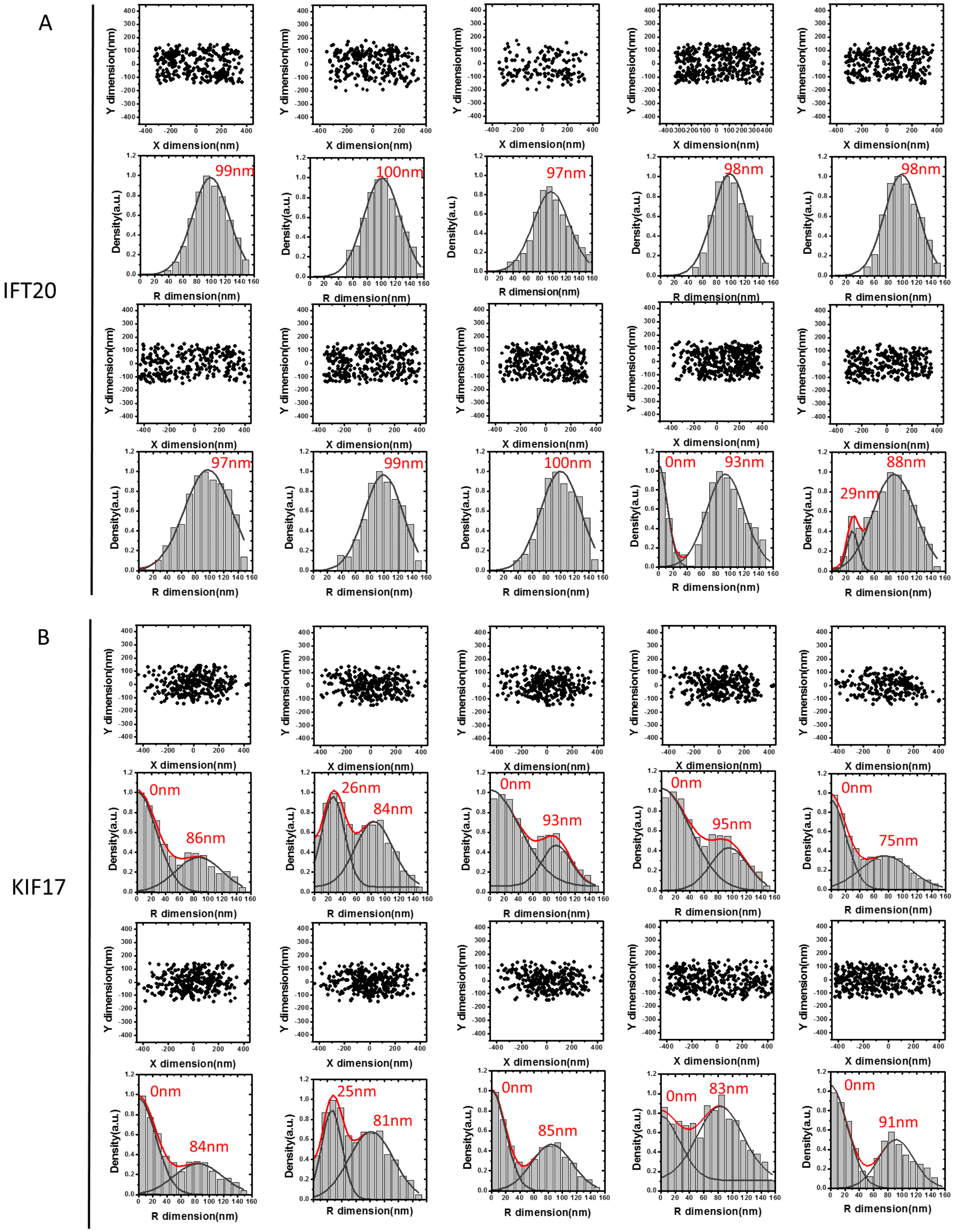
A
IFT20
Y dimension(nm)
X dimension(nm)
Density(a.u.)
R dimension(nm)
99nm
100nm
97nm
98nm
98nm
97nm
99nm
100nm
0nm
93nm
29nm
88nm
B
KIF17
0nm
86nm
26nm
84nm
0nm
93nm
0nm
95nm
0nm
75nm
0nm
84nm
25nm
81nm
0nm
85nm
0nm
83nm
0nm
91nm

Figure S3. List of 2D spatial distribution and the corresponding 3D spatial probability density distribution of IFT20 and KIF17 in ten different primary cilia of ten live cells. (A) 2D spatial distributions and the corresponding 3D spatial probability density distributions for IFT20 in ten different primary cilia. Gaussian function fitting was used to obtain primary locations (labels in red) for IFT20. (B) 2D spatial distributions and the corresponding 3D spatial probability density distributions for KIF17 in ten different primary cilia.

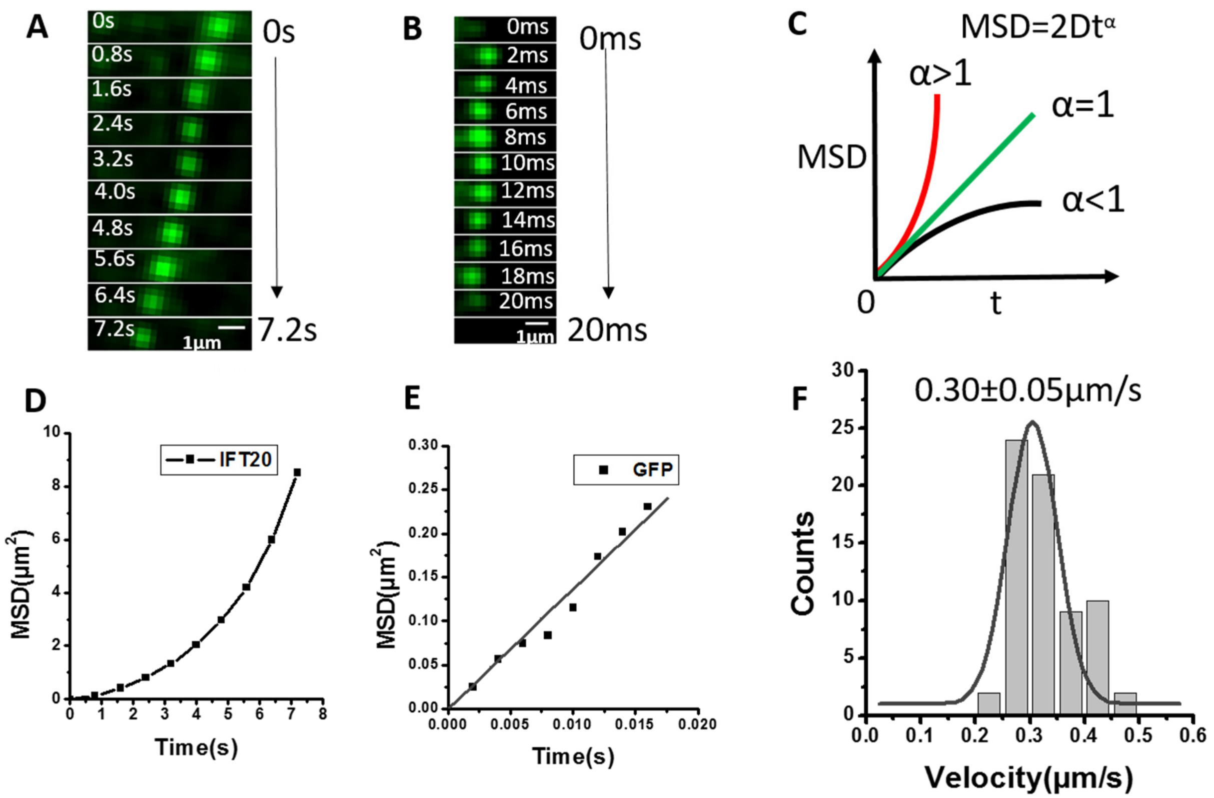

Figure S4 Determination of transport kinetics for IFT20 and GFP in live primary cilia. (A) As a demonstration, a single-molecule tracking event of IFT20-GFP for 7.2 s in live primary cilium is shown. Scale: 1 μm. (B) As an example, a single-molecule tracking event of GFP for 20 ms in live primary cilium is presented. (C) Typically the MSD plot is used to differentiate super-diffusion (or directional movement, α> 1), random diffusion (α=1) and sub-diffusion (α<1). (D) The MSD plot of IFT20-GFP traces as shown in A indicates that IFT20-GFP conducts directional movements in live primary cilia. (E) The MSD plot of GFP's diffusion in primary cilium as shown in B suggests that GFP passively diffuse in the axonemal lumen. An average diffusion coefficient of 3.6±1.7 $\mu m^2/s$ was obtained from one hundred and twenty diffusion events. (F) Based on sixty-two directionally moving events, an average velocity of 0.30±0.05 μm/s was determined for IFT20-GFP.

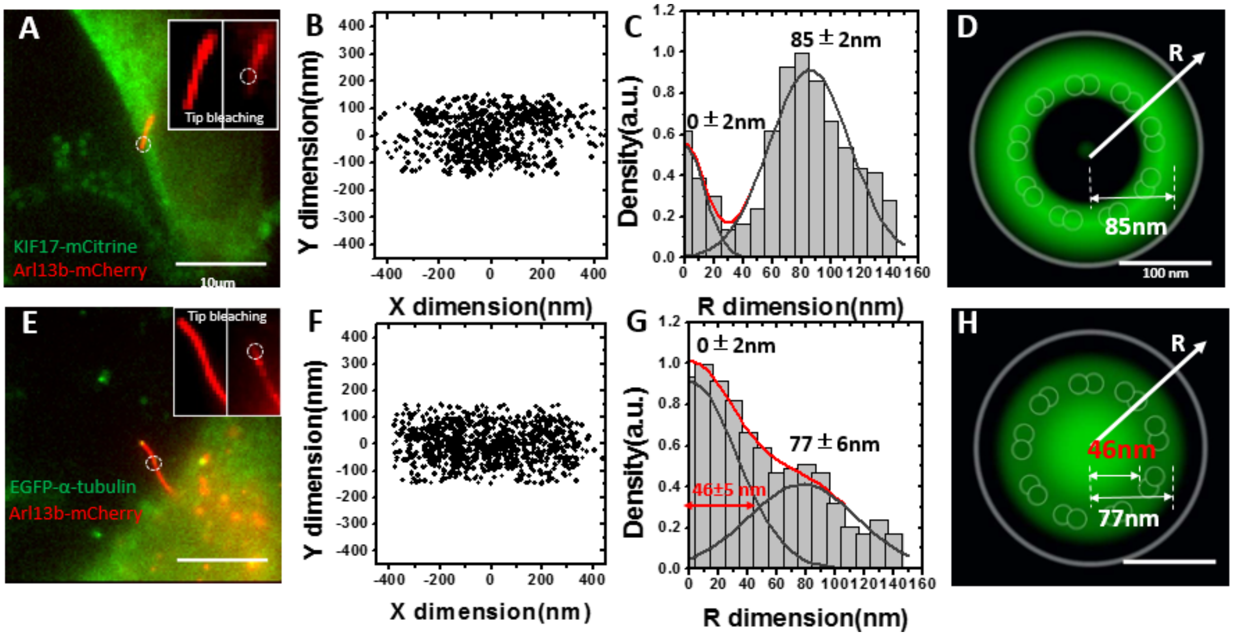

Figure S5 SPEED microscopy revealed that KIF17 and α-tubulin transport employ both the axonemal lumen and the IFT pathway in their anterograde transport in live primary cilia. (A) Representative image of Arl13b-mCherry and KIF17-mCitrine co-expressed in NIH3T3 cells. The circle denotes the laser illumination area of SPEED microscopy. The inset images show a primary cilium before (left) and after (right) photobleaching of ciliary tip. Scale bar: 10 µm. (B) 2D super-resolution spatial distribution of 710 individual KIF17-mCitrine locations collected from a single primary cilium. (C) By a 2D to 3D transformation algorithm, the spatial probability density distribution of KIF17-mCitrine inside the primary cilia along the R dimension was obtained. Based on Gaussian function fitting, two clusters of KIF17-mCitrine locate at radii of ~ 85 nm and ~ 0 nm in the cilia. The width of the central lumen route is ~ 50 nm, ranging from the very center to the crossing point of two Gaussian fittings. (D) Cross-section view of the spatial probability density distribution (green clouds) of KIF17-mCitrine in primary cilia, overlaid with the schematic in Fig. 2H. Scale bar: 100 nm. (E) Representative image of Arl13b-mCherry and EGFP-α tubulin co-expressed in NIH3T3 cells. The circle denotes the laser illumination area of SPEED microscopy. The inset images show a primary cilium before (left) and after (right) photobleaching of ciliary tip. Scale bar: 10 µm. (F) 2D super-resolution spatial distribution of 972

individual EGFP-α tubulin locations collected from a single primary cilium. (G) By a 2D to 3D transformation algorithm, the spatial probability density distribution of GFP-α tubulin inside the primary cilia along the R dimension was obtained. Based on Gaussian function fitting, two clusters of EGFP-α tubulin locate at radii of ~ 77 nm and ~ 0 nm in the cilia. The width of the central lumen route is ~ 100 nm, ranging from the very center to the crossing point of two Gaussian fittings. (H) Cross-section view of the spatial probability density distribution (green clouds) of EGFP-α tubulin in primary cilia, overlaid with the schematic in Fig. 2H. Scale bar: 100 nm.

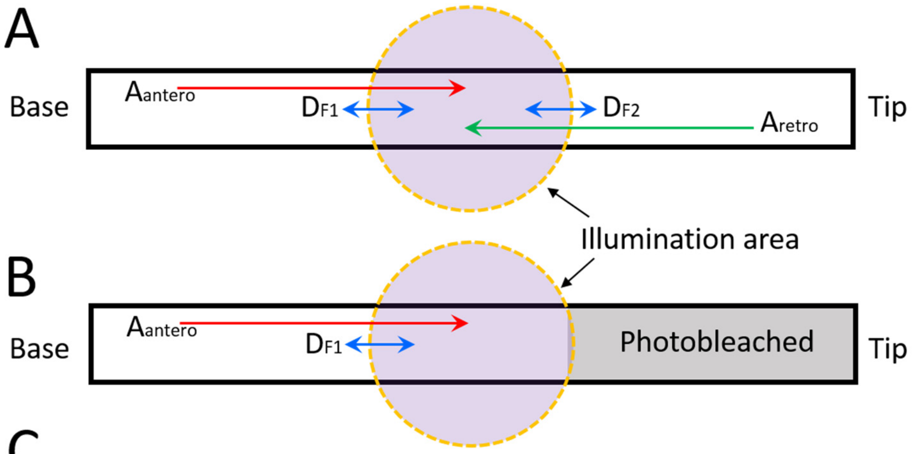

C

Given: $R1 = (A_{antero} + A_{retro})/(D_{F1}+D_{F2})$; $R2 = A_{antero}/D_{F1}$; $R3 = A_{retro}/D_{F2}$ (equation 1)

Based on: $A_{antero} + D_{F1} = D_{F2} + A_{retro}$ (equation 2)

Got: $R_3=(R_1R_2+2R_1-R_2)/(2R_2-R_1+1)$ (equation 3)

Probability: $P_{A_{antero}} = A_{antero}/(A_{antero}+D_{F1})=R_2/(1+R_2)$; $P_{D_{F1}} = 1- P_{A_{antero}}$ (equation 4)

$P_{A_{retro}} = A_{retro}/(A_{retro}+D_{F2})=R_3/(1+R_3)$; $P_{D_{F2}} = 1- P_{A_{retro}}$ (equation 5)

Figure S6 Determining the probability of using the IFT train pathway or the axonemal lumen route for protein candidates (KIF17 or α-tubulin) from the ciliary base to tip or vice versa. (A) In the illumination area (dashed circle filled in purple) of SPEED microscopy located at the middle of a non-growing primary cilium, protein candidates moving from the ciliary base and tip in either a pattern of directional movement (the IFT-train path) or passive diffusion (the axonemal lumen route) will be

captured. A_{antero}, A_{retro}, D_{F1} and D_{F2} denote the anterograde and retrograde transport rate, the diffusion flux of molecules coming from the ciliary base and tip, respectively. (B) Under the condition of completely photobleaching fluorescent protein candidates locating in half of cilium on the ciliary tip side, only A_{antero} and D_{F1} can be experimentally measured. (C) A group of equations was used to determine the probability (P) of using the IFT train pathway or the axonemal lumen route by protein candidates (KIF17 or α-tubulin) moving from the ciliary base to tip or vice versa, based on the ratio (R) of molecular spatial locations between the IFT-train pathway and the axonemal lumen route shown in the 3D maps of Fig. 3 and Fig. S5.

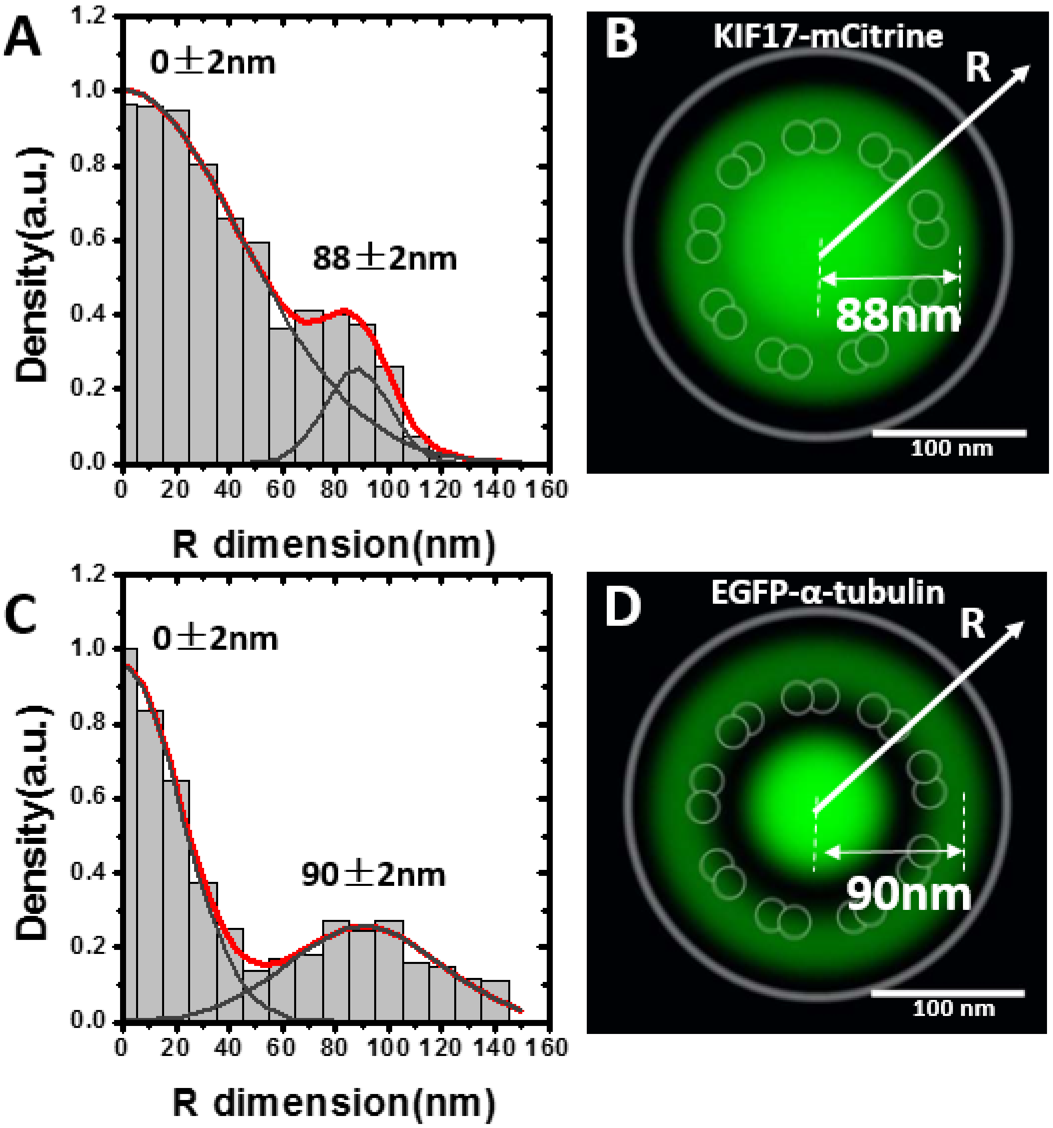

Figure S7 KIF17 and α-tubulin transport employ both the axonemal lumen and the IFT pathway when they move from ciliary tip to base in live primary cilia. As explained in Fig. S6, the results were obtained through subtracting the portion of moving from the ciliary base to tip (Fig. S5) from the summed 3D routes (Fig. 3). (A) The spatial probability density distribution of KIF17 inside the primary cilia along the R dimension. Based on Gaussian function fitting, two clusters of KIF17 primarily locate at the radii of ~ 88 nm and ~ 0 nm in the cilia, respectively. The width of the central lumen route is ~ 120 nm, ranging from the very center to the crossing point of two Gaussian fittings. (B) Cross-section view of the spatial

probability density distribution (green clouds) of KIF17 in primary cilia, overlaid with the schematic in Fig. 2H. Scale bar: 100 nm. (C) The spatial probability density distribution of GFP-α tubulin inside the primary cilia along the R dimension. Based on Gaussian function fitting, two clusters of GFP-α tubulin primarily locate at the radii of ~ 90 nm and ~ 0 nm in the cilia, respectively. The width of the central lumen route is ~ 80 nm, ranging from the very center to the crossing point of two Gaussian fittings. (D) Cross-section view of the spatial probability density distribution (green clouds) of GFP-α tubulin in primary cilia, overlaid with the schematic in Fig. 2H. Scale bar: 100 nm.

Reference for supporting information:

1. Ma J & Yang W (2010) Three-dimensional distribution of transient interactions in the nuclear pore complex obtained from single-molecule snapshots. *Proceedings of the National Academy of Sciences* 107(16):7305-7310.
2. Ma J, Goryaynov A, Sarma A, & Yang W (2012) Self-regulated viscous channel in the nuclear pore complex. *Proceedings of the National Academy of Sciences* 109(19):7326-7331.
3. Ma J*, et al.* (2013) High-resolution three-dimensional mapping of mRNA export through the nuclear pore. *Nature Communications* 4.
4. Ma J, Goryaynov A, & Yang W (2016) Super-resolution 3D tomography of interactions and competition in the nuclear pore complex. *Nature structural & molecular biology* 23(3):239-247.
